# Barriers and Facilitators for Implementing Paediatric Telemedicine: Rapid Review of User Perspectives

**DOI:** 10.3389/fped.2021.630365

**Published:** 2021-03-17

**Authors:** Louise Tully, Lucinda Case, Niamh Arthurs, Jan Sorensen, James P. Marcin, Grace O'Malley

**Affiliations:** ^1^Obesity Research and Care Group, School of Physiotherapy, RCSI University of Medicine and Health Sciences, Dublin, Ireland; ^2^W82GO Child and Adolescent Weight Management Service, Children's Health Ireland at Temple Street, Dublin, Ireland; ^3^Healthcare Outcomes Research Centre, Royal College of Surgeons in Ireland, Dublin, Ireland; ^4^Department of Pediatrics, University of California Davis School of Medicine, Sacramento, CA, United States

**Keywords:** telemedicine, telehealth, e-health, digital health, paediatrics, implementation

## Abstract

**Background:** COVID-19 has brought to the fore an urgent need for secure information and communication technology (ICT) supported healthcare delivery, as the pertinence of infection control and social distancing continues. Telemedicine for paediatric care warrants special consideration around logistics, consent and assent, child welfare and communication that may differ to adult services. There is no systematic evidence synthesis available that outlines the implementation issues for incorporating telemedicine to paediatric services generally, or how users perceive these issues.

**Methods:** We conducted a rapid mixed-methods evidence synthesis to identify barriers, facilitators, and documented stakeholder experiences of implementing paediatric telemedicine, to inform the pandemic response. A systematic search was undertaken by a research librarian in MEDLINE for relevant studies. All identified records were blind double-screened by two reviewers. Implementation-related data were extracted, and studies quality appraised using the Mixed-Methods Appraisal Tool. Qualitative findings were analysed thematically and then mapped to the Consolidated Framework for Implementation Research. Quantitative findings about barriers and facilitators for implementation were narratively synthesised.

**Results:** We identified 27 eligible studies (19 quantitative; 5 mixed-methods, 3 qualitative). Important challenges highlighted from the perspective of the healthcare providers included issues with ICT proficiency, lack of confidence in the quality/reliability of the technology, connectivity issues, concerns around legal issues, increased administrative burden and/or fear of inability to conduct thorough examinations with reliance on subjective descriptions. Facilitators included clear dissemination of the aims of ICT services, involvement of staff throughout planning and implementation, sufficient training, and cultivation of telemedicine champions. Families often expressed preference for in-person visits but those who had tried tele-consultations, lived far from clinics, or perceived increased convenience with technology considered telemedicine more favourably. Concerns from parents included the responsibility of describing their child's condition in the absence of an in-person examination.

**Discussion:** Healthcare providers and families who have experienced tele-consultations generally report high satisfaction and usability for such services. The use of ICT to facilitate paediatric healthcare consultations is feasible for certain clinical encounters and can work well with appropriate planning and quality facilities in place.

## Introduction

Telemedicine is an umbrella term for the use of information and communication technologies (ICTs) to facilitate remote consultations and deliver healthcare using computers and smart devices such as smart phones and tablet computers. Whilst the potential applications of telemedicine are all-encompassing, particularly in remote and underserved regions or for populations living with medical conditions for whom travel to healthcare appointments may be particularly burdensome, the emergence of the COVID-19 pandemic has significantly emphasised the need for secure ICT-supported healthcare. For healthcare delivery in particular, a need for safe alternatives to in-person care has rapidly come to the fore. During periods of rapid transmission of the virus, emergency department visits have sharply declined (1, 2) and routine screening and consultations have been virtually non-existent in many regions for long periods since the COVID-19 pandemic (3–5). This has resulted in a rapid and widespread increase in use of telemedicine and expansion of electronic healthcare to meet demand (6). It is likely that the need for infection control and social distancing measures will continue and may increase throughout the influenza and respiratory syncytial virus seasons. Reliable, secure, high-quality telemedicine will be vital for the continuation of healthcare services, particularly for those most vulnerable.

**Figure 1:**
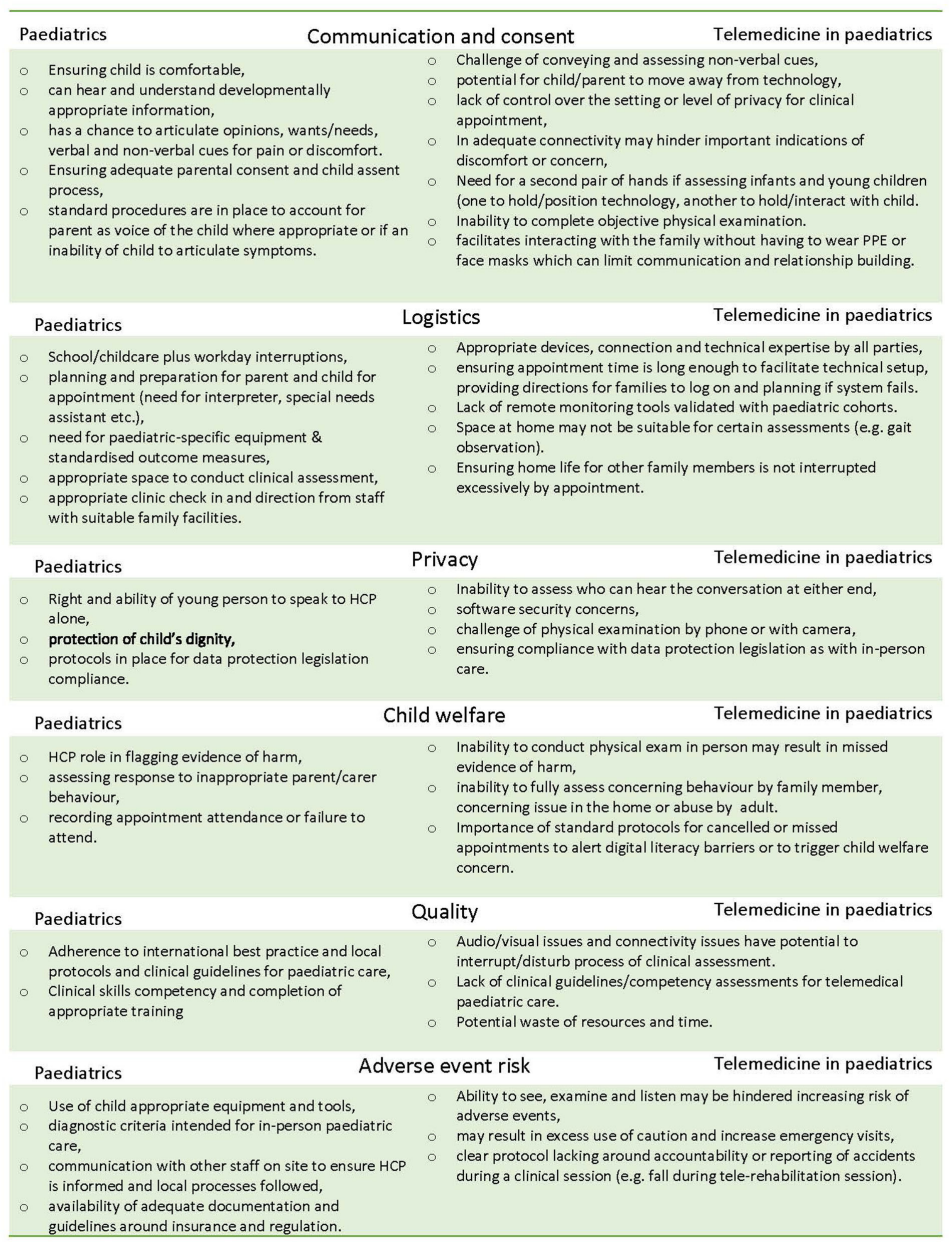
Special considerations for extending telemedicine to paediatric care.

Telemedicine for paediatric care warrants special consideration around logistics, consent and assent, child welfare and communication issues that may differ to adult services (Figure 1) (7). There is no systematic evidence synthesis available that outlines the implementation issues for incorporating telemedicine to paediatric services generally, or how users perceive these issues. We sought to identify factors that affect the establishment of virtual paediatric care in order to inform and equip those that need to urgently implement telemedicine (8), and assist paediatric service delivery in the longer term. Indeed, as noted by Ross et al. implementation does not stop with “go live” and therefore this review also informs those that have already implemented telemedicine (9). We aimed to achieve this by synthesising scientific studies that have documented barriers, facilitators, user attitudes and experiences of implementing paediatric telemedicine.

## Methods

We conducted a rapid systematic review (10, 11), using a concurrent mixed-methods evidence synthesis methodology (12). This review was registered on PROSPERO (registration number CRD42020184115).

A search strategy was developed and run in the MEDLINE database by a research librarian (Supplementary Image 1). We included any study examining aspects of implementing telemedicine for paediatric care, published in English between 2005 and 2020. This included studies whereby the technology facilitated paediatric consultations for patients and their caring adults. Studies were included if they assessed telemedicine undertaken in a clinical setting by healthcare professionals (HCPs) including physicians, surgeons, allied health professionals and nurses. References of relevant articles were also reviewed for eligibility. Full inclusion and exclusion criteria are available in the Supplementary Table 1.

All titles/abstracts and all potentially eligible full texts were screened by two of the three reviewers (LT and LC/NA). The reviewers discussed all conflicts and a consensus decision was made regarding inclusion. Data (study and participant characteristics, methods, findings consistent with the aims of this review) were extracted to Microsoft Excel and the Mixed-Methods Appraisal Tool (MMAT) (13) was used to assess the quality of included studies and risk of bias at outcome level. A randomly selected 20% portion of the extraction and assessment were independently verified (by LC/NA) to ensure quality.

Qualitative findings were coded (by LT) and analysed by the analytical themes identified from the developed code structure. We used thematic analysis, with guidance from Thomas and Harden (14). This process involves adding descriptive codes to the data and combining these to categorise the findings into themes using an iterative process. The identified barriers and facilitators were mapped to the constructs within the Consolidated Framework for Implementation Research (CFIR) (15), which involved categorising findings according to whether they are intervention-, individual-, setting- or process-specific (Table 1). Quantitative findings were summarised narratively.

**Table 1:** Summary of barriers and facilitators for implementation of telemedicine assessed qualitatively.

**CFIR construct**	**Barriers/challenges**	**Facilitators**
**Intervention characteristics:** Source Evidence strength and quality Relative advantage Adaptability Trialability Complexity Design quality and packaging Cost	• Lack of buy-in for need • Perception of additional work, complex, onerous • Uncertainty legality/credentialing • Fear of litigation • Lack of insurance coverage • Lack of confidence in the technology to be reliable • Fear of embarrassment (unreliable technology) • Outsider implementing programmes out-with perceived needs	• Perceived convenience, time & money savings for families • Perceived opportunity for learning • Straight-forward technology • “Plan B” protocols e.g., photos to complement poor video image
**Outer setting:** Patient needs and resources Cosmopolitanism Peer pressure External policies and incentives	• Misaligned incentives: loss of patients = loss of earnings • Perception that management get to “fly the flag” at any cost to staff	• Trust in providers ensures privacy
**Inner setting:** Structural characteristics Networks and communication Culture Implementation climate Readiness for implementation	• Implementation climate: perception of being tested or monitored • Fear of being replaced • Insufficient time/staff • Inadequate/no compensation • Paternalistic tone of remote colleagues	• Clear dissemination of telemedicine aims to all users • Reallocating administrative tasks away from those expected to use technology • Ability to offer wider services and thus better care • Calm and supportive tone among remote specialists • Equipment that fit into the environment • Strengthened relationships with outside teams
**Individual characteristics:** Knowledge and beliefs about the intervention Self-efficacyIndividual stage of change Individual identification with the organisation Other personal attributes	• Lack of familiarity between clinician and family • Lack of proficiency with technology • Working alone at home preventing interaction with colleagues • Reliance on subjective descriptions by parents & non-medical factors	• Having the option (for families) • Values: valuing effective care over reimbursement • Acknowledgement of cognitive bias which may influence decision-making
**Process:** Planning Engaging Executing Reflecting and evaluating	• Unclear aims goals of telemedicine service- inappropriate use	• Early comprehensive training, including communication training • Communication of the value of telemedicine—“selling it” • Allocated team time for debrief/reflecting with colleagues • Clarity on when to use telemedicine • Champions for telemedicine (for each discipline) • Accessible technical support • Appropriate triaging and referrals • Designating a suitable area for tele-consultations • Thorough planning and involvement of end users at all stages of planning and implementation

## Results

### Eligible Studies

**Figure 2:**
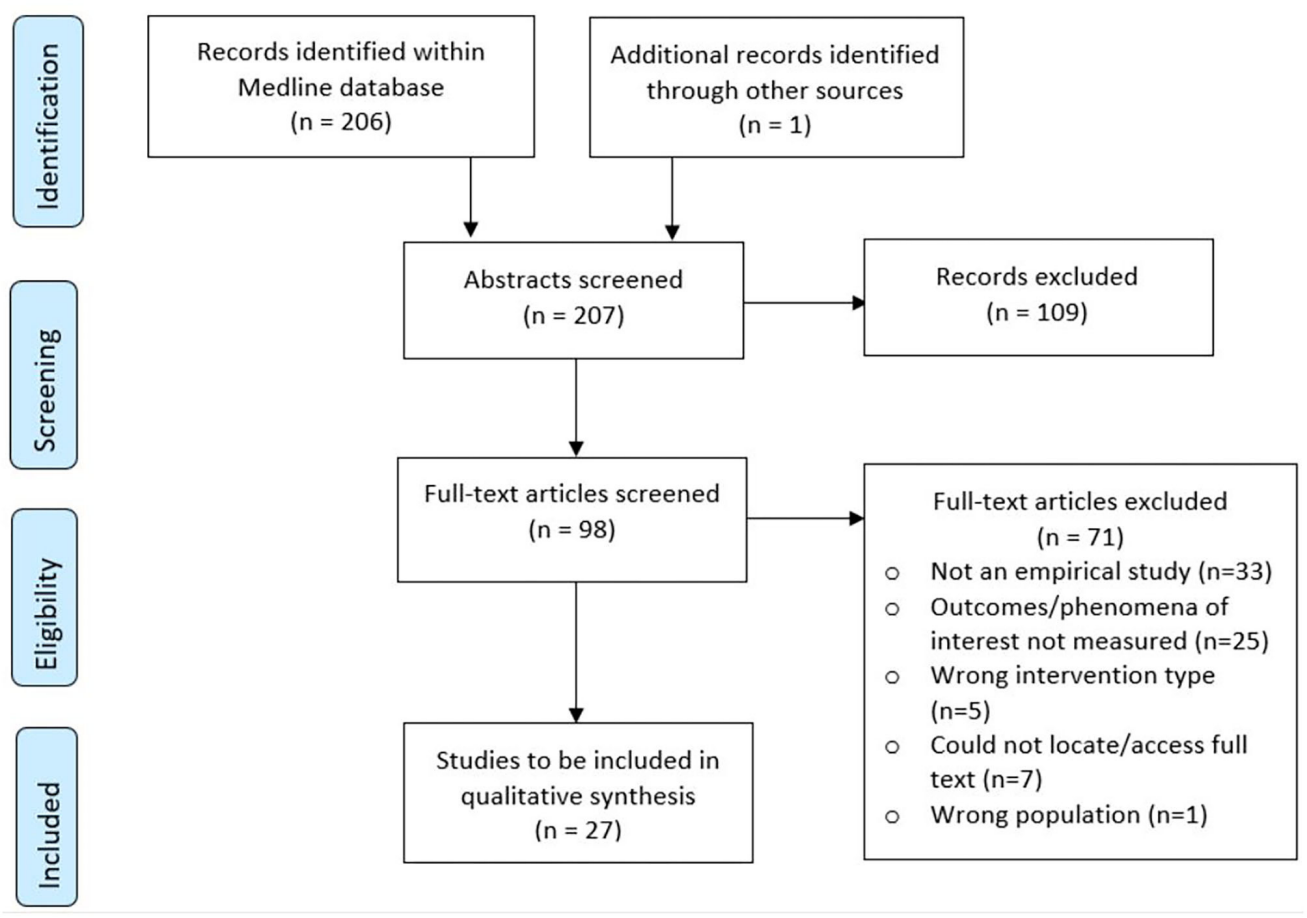
PRISMA flow diagram.

We identified 207 records in total from database searching and one additional title while scanning the references of the articles (Figure 2). Title and abstract screening excluded 110 records, while full text screening excluded 71. We identified 27 eligible studies; 19 quantitative studies (16 quantitative descriptive, two RCTs and one non-randomised trial); five mixed-methods studies, and three qualitative studies. All studies and their characteristics are listed in Supplementary Table 2. There was initially 86.4% agreement on screening decisions between reviewers (179/207 decisions), which increased to 100% agreement after discussion.

### Quality Appraisal

The full quality appraisal results, as presented according to the MMAT items, can be seen in Supplementary Table 3. To briefly summarise the quality of included studies, most quantitative descriptive studies (which represented 16/27, 59% of the included studies) were generally moderate to low quality. The primary reason for low scores was ambiguity or low quality relating to the instrument used for assessing attitudes/experiences among participants (i.e., the tool used, its development, validity or reliability, appropriateness within the specific setting), in addition to unclear reporting of response rates or whether the samples surveyed were representative. Three trials (16–18) were of high quality. However, the study by Cady et al. (16) only assessed the outcomes of interest for this review as open-ended feedback post-intervention. The mixed-methods studies consisted of two high quality papers and three lower quality. One study scored low based on an unclear research question and thus inability to assess whether the design was best placed to answer it, while two scored low due to insufficient detail presented for assessment of the qualitative components. The three qualitative studies were generally of high quality.

### Qualitative Synthesis

The themes identified from the qualitative and mixed-methods data are described below. Table 1 summarises the barriers and facilitators for implementation of telemedicine as presented within these themes, according to the domains of the CFIR framework.

#### Buy-In

Several issues were described relating to participant buy-in for the use of telemedicine as an alternative for in-person paediatric care, or as a tool for accessing specialist care remotely. Among HCPs, buy-in to the benefits of and need for telemedicine was an important facilitator for its uptake and use (19), and there was apprehension expressed by some providers about its introduction to paediatric services (20). Uscher-Pines et al. reported that HCPs believed that video conferencing was being proposed for cases whereby a “phone call would suffice,” adding additional work and unnecessary complexity (19). Other barriers were related to the perception that they were being tested or monitored, or that it would increase the potential for having their decisions questioned (19, 20), specifically whereby the telemedicine service was between a remote site and a specialist hub. Participants in one study (20) proposed increased reassurance to staff that these were not the aims of the telemedicine service, in order to increase uptake and buy-in (21).

If providers suspected that the use of telemedicine would be onerous, complex or that the technology would be unreliable, they were less likely to use it according to one study (21). Initiating care through telemedicine without previous familiarity of a family/case was also cited as a concern among providers (22). Participants suggested various strategies for facilitating buy-in including early comprehensive training in the technology to increase comfort with its use, accommodating time for implementation by redirecting other time-consuming tasks away from busy providers (19), and communicating the value and potential benefits widely to potential users in advance (19, 23). Some patients and families had reservations about tele-consultations with unfamiliar clinicians, or those with whom they did not have a relationship. Choice between telemedicine and face-to-face care was a suggested facilitator for buy-in among families (24).

“*I would like to think that this is something that is going to be a part of the care, not is going to become the norm. So that would bother me, because I think it's still important to be able to have that option to come in and have your child seen, vs. ‘Oh, I think if we just do a conference call we're fine.' I don't…I'd like to see, you know—I don't know. That would just be a concern of mine”* (24).

#### Financial, Regulatory, and Legal Considerations

Concerns were raised by HCPs across multiple studies around the legality of care using telemedicine. One study reported that providers had serious reservations about telemedicine due to their inability to assess risk in paediatric patients the same way they could during an in-person visit, in addition to the risk of a misdiagnosis, resulting in a fear of litigation arising from its use (22). This fear influenced HCPs' decisions made via telemedicine.

“*Everything was documented since I had more concern in this work about lawsuits. The documentation was very detailed and meticulous. There were those I would return to after a few hours. the inability to examine closely certainly influenced, and it is difficult to make decisions in this consultation. I did not feel confident enough to make decisions…”* (22).

The issue of credentialing, the process of ensuring legitimacy of care through the medium of telemedicine, was discussed in detail and described as onerous and time-consuming (19). A variety of interpretations of the need for specific credentialing for telemedicine was reported across different sites, which varied from this being a barrier for uptake due to local laws, to some sites concluding that no additional credentialing was necessary (19).

Karlsudd et al. reported that, where families waived their right to confidentiality, it facilitated a more open exchange of information and allowed for efficiency in terms of multi-disciplinary care (25). From the perspective of the patient/family, parents had little concern related to privacy, though did report hoping it was well-managed by the healthcare organisation (24).

Uptake of telemedicine among families was found to depend heavily on whether insurance companies were willing to reimburse care by this means (24). One study found that the administrative time spent organising billing for telemedicine was reported to be too time consuming, and that lack of insurance coverage in addition to inadequate reimbursement for tele-consultations were perceived to be major barriers for the long-term sustainability of telemedicine (19).

#### Relative Advantages vs. Opportunity Costs

The advantages of telemedicine for patients and families were widely recognised to include time saved by avoiding travelling to appointments (25), with the consequential effect of reduced absenteeism from school for patients and work for parents/carers (24, 26), reducing stress and burden for families (19, 24). This was reported to result in financial savings for families also, related to travel and associated expenses (26). Some observed benefits went much further than convenience however, with the implementation of telemedicine allowing for access to appropriate and timely specialist care for children far beyond what had previously been available, particularly in remote areas (19, 20, 24, 26). HCPs who participated in one study expressed relief at the enhanced capacity that telemedicine allowed for (26).

The same study found that rural families saw the ability to connect with tele-psychiatry and its benefits as an opportunity to become active members of their community again. Families expressed a sense of hope as a direct result of the implementation of this service, with a suggestion that this could even contribute to the stability of rural communities. For children with chronic illnesses, it was reported that telemedicine was viewed by families as offering the potential to streamline access to multi-disciplinary care and also reduce the risk of cancellation of appointments due to illness.

“*There are times when she's too weak to get up, and I've had to cancel appointments. Instead of cancelling, I would have loved to have had the ability to say, ‘Hey, she can't get up today. I don't want to cancel. Here you know, let's video-conference and discuss what's going on'…”* (24).

Ray et al. also reported that families expressed feeling that telemedicine would allow for reassurance and reduced anxiety about a child's condition between in-person hospital visits, and could also allow for more logical/efficient scheduling for healthcare, one example given being a screening/triage system to assess need for an in-person visit, and therefore increase the value of in-person care (24).

#### Change Management

In contrast, however, telemedicine was widely reported to be additional work on a practical level from the perspective of HCPs, and in particular its implementation tended to involve what staff perceived as excess paperwork/administrative tasks (19, 20, 27). This was compounded in cases by ICT illiteracy resulting in tasks being completed manually by those not proficient with the software (27). Some HCPs added that using telemedicine, which often meant working out of their own homes, was sometimes isolating and that the inability to run cases, issues and ideas past colleagues in the clinical environment was a drawback (22). In some cases, these issues were expressed with frustration that this work came without additional compensation, although other providers acknowledged feeling that the ability to provide effective care was more valuable than reimbursement (19).

On a more profound level, providers also expressed concerns around the broader pathways associated with implementation of telemedicine, whereby offering a one-time consultation would not be a solution to patients for whom there was a dearth of access options (26). Participants in another study expressed apprehension around misaligned incentives also, within a jurisdiction whereby healthcare provision is often for-profit, and therefore losing patients equated to loss of earnings/income and so, introducing telemedicine for remote care was not always in the interests of everyone involved (19). Haimi et al. on the other hand found that in some cases providers did not view saving money for the healthcare service/system to be a priority when considering the use of telemedicine (22).

#### Impact on Quality of Care

The use of telemedicine was reported to both positively influence, and at times hinder clinical decision-making among providers. The support of specialist input to satellite healthcare providers for instance, was found to instil confidence and reassurance in the ability of local providers to give appropriate care (20, 26). In some cases however, the fear of having their clinical judgement questioned or having a decision overturned as a result of using the telemedicine service was a barrier to uptake of the service (19).

Some clinicians discussed how telemedicine could not replace in-person consultations with families, and this was a source of apprehension about its use. Others were reassured that video allowed for an opportunity to provide care rather than nothing/only a phone call, despite being seen as inferior to in-person care (22). Among those who were less confident in their ability to make judgements via telemedicine, the worry of children's inability to express symptoms, in addition to frustration at being unable to gather sufficient information whilst under time pressure given the acute nature of paediatrics, was described as being a primary source of worry. Many participants discussed their need to rely on subjective descriptions provided by parents (22). This was echoed by parents in another study who felt under pressure to provide accurate descriptions of their child's condition and feared they would not convey all the necessary information, which increased their anxiety about the process (24).

“*I suppose the fact that they can't really see him, I guess, and if I can't really say for sure what's wrong with him…if I couldn't explain what's going on with him, I might make it sound not as bad as it actually is or I might make it sound worse”* (24).

In contrast, other parents saw telemedicine as an opportunity for better access to care and timely diagnoses (24), though from a provider perspective, some talked about the conflict of “good service vs. proper medicine,” whereby they felt the need to oblige parents who misused the telemedicine service for convenience (22).

Healthcare providers interviewed by Haimi et al. discussed the non-medical factors they relied on to help guide decisions where needed, and these included parents' tone of voice, perceived health literacy of the parent and their perceived ability to make shared decisions with the family. Some participants acknowledged the need for awareness of their own cognitive biases that may affect judgement in such circumstances, an example of this being the perception of a family's socioeconomic status, which participants cited as one factor considered when making decisions using telemedicine (22). The same study found that younger physicians, and those who had studied medicine in less “conservative or patriarchal” cultures tended to be better able and more open to shared decision-making with families.

#### Reliability and Usability of Technology

Issues with the usability and complexity of the technical platforms for facilitating telemedicine were widespread across studies. Their quality, reliability and the proficiency of clinical users were major factors in determining its acceptance and uptake among staff (19, 22, 27, 28), and some families (24). Participants discussed connectivity issues reducing their utilisation of telemedicine (27), with long setup times, audio-visual issues (21, 22), and “background fears” of something going wrong constantly affecting the quality of a consultation (20). Some clinicians described feeling embarrassed by these issues, which were often beyond their control. This issue was not unique to older studies, with the issue observed in those published up to 2018.

“*Equipment can be hard to use and it looks like you don't know what you are doing to the person on the other end. It is an ongoing challenge to keep people competent when volume is low”* (19).

Insufficient training on the telemedicine equipment/technology was a reported source of technical problems in the same studies where ICT illiteracy was a cited major barrier to uptake of telemedicine (19, 27). Other interviewees however noted that confidence with the technology grew with increased use and experience of tele-consultations (22). Some clinical staff made suggestions for potential facilitators for smooth implementation, including having the facility for families to send photos when video quality was insufficient (22) and ensuring access to all necessary medical records via the telemedicine software (28). Participants also suggested investment in user-friendly equipment that fit well with the existing clinic, in addition to continued staff training (19, 20), availability of technical support (28), and frequent testing of the equipment by staff outside of scheduled consultations (19). It should be noted that among participants who found their telemedicine platform to work well, improved communication between families and clinical staff was reported, in addition to allowance for “genuine further education” (25).

#### Integration to the Organisation

Healthcare providers described the implementation of telemedicine as having allowed for streamlining of care processes, which had a positive impact on care (20). Appropriate triaging, appropriate referrals for telemedicine consultations and practicalities such as having a suitable area for staff to carry out tele-consultations comfortably were all cited as facilitators for its use (28).

Where clinical staff reported feeling less satisfied with the integration of telemedicine to the local workflow, these issues tended to be around how expectations and logistics had been managed (19). Participants conveyed dissonance between management and staff, describing the impression that telemedicine was implemented as a tick-box activity for the organisation, without careful planning.

“*The [hub] hospital gets to wave the flag that they offer this service, but the [hub] doc just has to work harder for no additional compensation”* (19).

Insufficient staff numbers with capacity to engage with patients via telemedicine was a problem encountered by others (20, 22), which prevented use of the service.

Beyond individual settings, the implementation of telemedicine was also described by some to facilitate strengthening of relationships between clinical sites (19) and disciplines (25), and where calm and supporting communication was used for tele-support between sites, this facilitated acceptance of this service (20). In contrast however, the use of overly paternalistic tone of communication by remote specialists was a barrier to engagement cited by satellite staff (20).

Suggestions for facilitating integration of telemedicine services to existing organisations arising from these discussions were common. Thorough planning with consideration for each aspect of implementation, logistics and administration as well as cultivation of clinical champions across the relevant disciplines within the healthcare setting were suggested (19). Additional suggestions included allocation of staff to coordinate and support telemedicine and its various tasks (20, 28), involvement of frontline staff within the organisation throughout the implementation process (20), a designated clinician to accompany patients at remote facilities (28), and additional support for ensuring follow-up and adherence to patient recommendations arising from the tele-consultation (26). Finally, need for clear dissemination of the purpose of telemedicine to ensure appropriate use, and allocated time online with peers for those working in isolation to reflect, debrief and discuss their experiences were described (22).

### Quantitative Synthesis

#### Attitudes to Telemedicine vs. Usual Care

Four studies assessed attitudes to telemedicine as an alternative to in-person visits, among families who had not yet experienced telemedicine and found high (95%, 151/159) (29) to moderately high (58% 148/256; 57%, 588/1032) (30, 31) preference for in-person visits, despite openness to trying telemedicine (30, 32). For studies whereby telemedicine had been tested (18, 33–37), acceptability of tele-consultations ranged from 79 to 100%. Qubty et al. also reported feedback that telemedicine is useful if the child is doing well, otherwise face-to-face is preferable (34). Marconi et al. examined physician tele-presence during an emergency triage and found that 59% of parents and 83% of children would prefer this type of visit (18).

Time/distance spent travelling to appointments (29–31), perceived cost of in-person appointments (31), familiarity with telemedicine (31), and number of missed work hours (38) were all significantly correlated with positive attitudes to telemedicine.

#### Usability

Of the five studies that reported usability from the perspective of HCPs, the majority found the technology easy to use (90%; 95%) (20, 39) or rated it highly (9.3/10.0; 4.2/5.0) (25, 40). Zachariah et al. reported all clinicians to be competent with independent use of telemedicine following training on use of the equipment (35).

Among patients and families (*n* = 1,032), one study found participants to be comfortable communicating about medical issues through email (69.9%, *n* = 721), telephone (82.9%, *n* = 856), and video conferencing (52.9%, *n* = 546) (31). Others reported unanimous satisfaction and comfort with the experience of using telemedicine (98%; 100%) (33, 34), and high ratings for user-friendliness of the telemedicine platform (4.8/5.0) (25).

#### Challenges Encountered

Table 2 presents the main barriers to initiating use of telemedicine that were reported across six quantitative studies. The challenges encountered with the use of telemedicine that were reported quantitatively by seven studies are shown in Table 3.

**Table 2:** Reported barriers to initiating the use of telemedicine.

**Author**	**Perspective**	**Barrier**	**Frequency reported**
Fieleke	Healthcare provider	Lack of need Billing/reimbursement issues Concerns about medico-legal ramifications Lack of trust in telemedicine accuracy Lack of direct patient contactCost Time constraints	Twice or more
Fang	Healthcare provider	Lack of clinical need	65·5% (36/55)
McCrossan	Healthcare provider	Insufficient training in relevant specialty	87% (13/15) of those using telemedicine infrequently (37% in total, 13/35)
		Inexperience with the equipment	31% (11/35), (73% in total, 11/15)
Seckeler	Healthcare provider	Patient privacy concerns	60% (27/46)
		Cost of implementation	10% (4/46)
		Ease of access in the catheterization laboratory	10% (4/46)
		Image quality	10% (4/46)
		Time constraints	10% (4/46)
		Trust of advisor (technology for communication with mentors)	10% (4/46)
Russo	Patient/family	Lack of trust toward telemedicine tools	30%
		Fear of excessive responsibilities for the family	28% (of those who expressed non-interest in telemedicine; n = unclear)
Marconi	Patient/family	Child too sick to take part	Most common reason for declining to participate; % not reported

**Table 3:** Reported challenges encountered during use of telemedicine.

**Author**	**Perspective**	**Issues reported**	**Frequency reported**
Brova	Healthcare provider	Process concerns	39% (42/107)
		Technology concerns	14% (15/107)
Fieleke	Healthcare provider	Poor image qualityPatient movement leading to blurred imagesInability to perform necessary examination/treatmentBilling/reimbursement issues	Twice or more
Hopper	Patient/family	Perception that telemedicine examination was insufficient	10% (1/10)
		Child distracted/bothered by screen	10% (1/10)
McConnochie	Healthcare provider	(Reasons for incomplete visits)	
		Inability to perform necessary examination/ treatment remotely	64% (51/79)
		Further test or imaging needed	14% (11/79)
		Child site or parent decision prevented clinician from seeing child	4% (3/79)
		Technical failure/inadequacy	17% (14/79)
		(Reasons for cancelled/refused visits)	
		Designated clinicians for tele-consultations out of office without cover	40% (96/243)
		Practise indicated being too busy to accommodate tele-visit	19% (47/243)
		Insurance did not cover telemedicine/no insurance	18% (43/243)
		Visit requested too late	11% (27/243)
		Administrative error/issue unrelated to the technology	3% (7/243)
		Practise unable to complete visit within available time	2% (4/243)
		Practise refused visit due to unpaid bill	<1% (1/243)
		(Reasons for abandoned visits)	
		Parent picked up child before information capture was complete	25% (23/90)
		Unable to acquire necessary information (e.g., child uncooperative)	15% (14/90)
		Administrative problem (e.g., unable to contact parent for consent)	20% (18/90)
		Technical problem	12% (11/90)
		Problem was beyond capacity of model	10% (9/90)
		Other (not specified)	18% (15/90)
Qubty	Patient/family	(from open feedback) Sub-optimal audio/video Connectivity issues due to capacity of home internet service Not optimised for tablet PC Insufficient troubleshooting resources for families Telemedicine calendar not open early enough to find available slots Administrative burden No sign interpreter	26% (13/51) 8% (4/51) 2% (1/51) 4% (2/51) 2% (1/51) 4% (2/51) 2% (1/51)
Seckeler	Healthcare provider	Encountered inadequate imaging to provide advice	42% (8/19)
Zachariah	Healthcare provider	Temporary disruptions in audio (sound distortion) and video (image streaking) quality requiring widening bandwidth of the internet provider.	86% (6/7)

Participants within some studies offered suggestions for improvements of telemedicine services. These included the need for training and education (17%, 7/41; 100%, 7/7), and suggested investment in higher quality equipment with higher resolution imaging (7%, 3/41; 100%, 7/7) (35, 41). Fefferman (40) reported no negative feedback, while Brova et al. reported 39% (42/107) to have experienced no significant implementation challenges. No studies reported whether any adverse events related to the use of telemedicine occurred and no detail was provided within the included trials about whether this was monitored (16–18).

#### Perceived Benefits of Telemedicine

Table 4 outlines the perceived benefits of telemedicine. Time-savings were cited across more studies than any other beneficial factor, with eight papers reporting that it was mentioned. One additional study (32) found that most respondents thought that time-saving was moderately/very important (88%), followed by cost-saving (85%) among those who had not yet tested telemedicine.

**Table 4:** Benefits of telemedicine as perceived by participants.

**Benefit cited**	**Study**	**% (*n*)**
Time savings	Fefferman^a^	-
	Fieleke^a^	-
	Lai^b^	24% (5/21)
	Qubty^b^	85% (4/51)
	Seckeler	82% mentors (16/19); 65% (30/46) mentees
	McConnochie	91% (207/227) (mean saving 4·5 h; SD 2·2)
	Cady^b^	1% (2/139)
	Karlsudd^c^	-
Increased efficiency	Fefferman^a^	-
	Fieleke^a^	-
	Cady^b^	4% (5/139)
Convenience	Fefferman^a^	-
	Lai^b^	10% (2/21)
	Qubty	100% (51/51)
	Cady^b^	2% (3/139)
Lower cost	Lai	10% (2/21)
	Qubty	100% (51/51)
Increased communication/ familiarity/ solidarity between staff/services	Fefferman^a^ Seckeler Zachariah Fang Karlsudd^c^	– 82% (16/19) mentors 71% (5/7) 90% (84/93) –
Improved workflow/patient management/protocols	Fefferman Fieleke^a^ Zachariah Fang Karlsudd^c^	100% (16/16) – 86% (6/7) 85% (79/93) –
Increased learning opportunities	Fefferman^a^ Fieleke^a^ Zachariah	– – 100% (7/7)
Improved enjoyment of visits for paediatric patients	Fieleke^a^	–
Reassurance (for professional or parent)	Lai Cady^b^	14% (3/21) 1% (1/139)
Reduced stress	Qubty^b^	2% (1/51)
	Cady^b^	1% (1/139)
Reduced risk of infection	Cady^b^	1% (1/139)

a *Open-ended feedback, frequency not reported*.

b *Open-ended feedback*.

c *Presented as average scores out of 5.0 (parents/staff): time savings (4.6/3.5); synergy effects (4.6/3.4); increased quality of contact and information (4.5/3.5)*.

#### Satisfaction With the Telemedicine Service

Overall satisfaction with telemedicine was reported among six studies that assessed the patient/family perspective (16, 17, 25, 33, 34, 36), with two of these as part of randomised controlled trials (16, 17). Coker et al. (17) found that parents reported significantly higher satisfaction with a tele-referral system and with care overall compared with usual care. Cady et al. (16) reported significantly higher “adequacy of coordination of care” among participants within the intervention group of a three armed trial testing phone, video and usual care, compared to baseline. No significant differences were observed between groups. Four studies reported high satisfaction with telemedicine care received (25, 33, 34, 36).

HCPs' satisfaction with telemedicine was reported quantitatively by eight studies (20, 25, 35, 36, 39, 40, 42, 43), with generally high satisfaction ranging from 91-100% among those whereby the telemedicine was used for communication with patients/families (20, 35, 36, 39). McConnochie (42) found that 46% were at least as confident of diagnoses made via telemedicine as face-to-face. This increased to 83% among providers who had carried out over 50 tele-consultations. High satisfaction with technology for communication between professionals was also reported (40, 43). Karlsudd et al. reported greater satisfaction among parents (4.8/5.0) than HCPs (3.9/5.0) (25).

## Discussion

### Summary of Findings

We aimed to identify and describe the scientific literature related to implementing telemedicine in paediatrics. This study is essential as it informs and supports the response of paediatric health services to the COVID-19 pandemic and the efforts needed to maintain clinical services while adhering to pandemic-response guidelines. We present a synthesis of evidence for factors affecting implementation of paediatric telemedicine from the perspectives of end-users, including HCPs and patients/families. In addition, we map the findings to the CFIR (Table 1) to facilitate systematic identification of multi-level factors reported to influence implementation of telemedicine in paediatrics. The use of CFIR provides readers with a practical guide allowing stakeholders to apply relevant findings to their own paediatric setting. Our review provides an outline of the broad issues that have been identified within a set of studies of variable quality, settings and clinic types, informing actionable considerations for current implementation plans whilst also providing evidence to inform further primary research and focused evidence syntheses. This review also collates evidence for both paediatric patient/family and HCP acceptance of telemedicine for the first time.

The quantitative studies assessed demonstrate that among those who have not yet tried telemedicine, there was a tendency to favour in-person care, however among those who had tested tele-consultations, acceptance and satisfaction was high, increasing also with experience. Families who lived further away from healthcare facilities, and who therefore had greater costs (both monetary and opportunity costs) for attending in-person appointments, were more open to tele-consultations. This is of particular importance in paediatrics whereby both school and workdays are potentially missed due to healthcare appointments.

Several barriers to uptake and challenges were identified within the quantitative literature specific to paediatric care and telemedicine generally, and scepticism about the reliability of the technology was a key barrier expressed by both providers and families. Telemedicine was perceived as inappropriate for various types of examinations logistically, and often could not replace in-person visits, while other common challenges included connectivity and quality issues, specifically inadequate audio/visual quality. Many of these issues were echoed by the qualitative studies, where it was also clear that HCPs experienced a great deal more practical issues and concerns around the use of telemedicine than patients and their families, who valued the convenience it allowed. Thorough planning before implementation commencement and involving frontline staff in order to identify practical concerns within a specific setting and to increase buy-in, is a key finding. Investment in quality, reliable technology that staff can trust to overcome the communication considerations for working with families, in addition to appropriate reallocation of resources to allow the service to run and comprehensive training are also necessary. For paediatric care specifically, a key consideration is the importance of triaging patients for the suitability of telemedicine (e.g., whether a tele-medical consult might expedite access to specialist care, whether a physical assessment can feasibly be undertaken without physical examination or whether physical rehabilitation can occur without therapeutic handling). Secondly, the inability of children to describe and express symptoms depending on age/development should be considered and is of particular importance *in situ*ations where child welfare may be at risk. Thirdly, with young children, there can be difficulty in capturing images electronically, which in addition to general anxiety among staff using telemedicine, impacts decision making and can result in additional caution.

### Previous Literature

Many of our findings are consistent with those outlined by reviews of telemedicine in broader populations, for many of the aspects of implementing telemedicine generally (9, 44). Concerns about liability and reimbursement were also raised in a review of statutes and regulations for telemedicine for stroke care in the U.S. (45) and this was prominent with our review, particularly among clinicians in the U.S. Costs and reimbursement issues were further highlighted by Helleman et al. in a review of tele-care for amyotrophic lateral sclerosis (46), who also reported evidence of perceived benefits that were closely aligned with the findings of this review; continuity of care, convenience, time-savings and reduced travel burden. Concerns among clinicians about lack of opportunity to conduct a physical examination and the resulting limitations on care were also emphasised (46). Our review, however, is the first to synthesise the evidence for barriers and facilitators for implementing telemedicine in paediatric settings and highlights additional considerations pertinent to paediatric care. For example, the inability of younger children or those with communication difficulties to describe their symptoms requires interpretation by carers and HCPs. Such assessment and interpretation may not be as easily conducted through tele-consultations. Secondly, taking informed parental consent and child assent using tele-consultations may be challenging. This adds additional pressure to both parents/carers and clinicians to accurately assess the level of risk associated with the child's condition and act accordingly, and may result in decreased confidence in the use of the telemedicine medium for paediatric care compared with adult care.

### Considerations and Future Research

In addition to the findings of this review, further considerations for the context of urgent implementation of telemedicine as a response to a global pandemic are needed. The absence of in-person care may greatly infringe upon the ability of HCPs to identify issues relating to child protection such as compliance with immunisation schedules or evidence of potential harm, particularly in regions whereby schools may close during the COVID-19 pandemic and the opportunities to flag such issues are greatly reduced. Concerns about assessment of risk were highlighted within this review (22). However, the broader assessment of risk within the context of child welfare in the home is another role of the healthcare provider (47, 48) and emphasises the balance needed between maintaining care via telemedicine while no alternative is available, while monitoring and evaluating its feasibility as a long-term replacement for in-person care. Our review also highlights the dearth of data related to the reporting of adverse events in tele-medical interventions and future studies should ensure such data is collected and reported.

In many cases the planning, staff consultations, time, and funding necessary for gold standard implementation will simply not be available, while additional necessities such as staff working from their own homes and related privacy issues must also be considered. From the perspective of families, the need for quality technology and connectivity may contribute to issues of inequity and could increase socioeconomic disparities. Recently documented issues have emphasised security concerns however (49) and particularly in Europe, compliance with General Data Protection Regulations (GDPR) is the primary criterion for selecting appropriate platforms. Monitoring and evaluation of implementation that occurs, over the course of the pandemic and beyond, will offer insight into barriers and facilitators of rapid implementation in the context of a pandemic. It is important that well-designed process evaluations and assessments of user-experiences are undertaken, with meaningful data captured in order to inform future service design and optimise the capacity for using telemedicine safely and effectively.

### Strengths and Limitations

This study had several strengths and limitations due to its nature as a rapid evidence synthesis. We searched one database due to time constraints, in the interest of producing a review of the key issues in a timely manner to be of maximum use. As a result, we have not assessed the breadth of the available literature on this topic for this review. Additionally, this review covers a variety of studies, which are heterogeneous in terms of the technology used, the clinical setting observed, a mix of high, low and middle income countries, and having been undertaken over a period of 15 years. Technological issues described by older studies may no longer have relevance in countries where IT infrastructure has rapidly evolved. However, many of the articles identified for inclusion still produced insightful comparable data on implementation. This article provides an overview of aspects of implementation of paediatric telemedicine that future research can build upon through carefully planned, robust and exhaustive reviews with more tightly focussed inclusion criteria.

Our inclusion of multiple research methods however, allowed for a comprehensive and rich overview of the factors involved in paediatric telemedicine. We undertook steps to minimise risks of bias, including double screening of records and verification of quality appraisal and data extraction by additional members of the review team. While the quality of the included quantitative literature was not consistently high, this highlighted a need for comprehensive feasibility studies that incorporate implementation fully into their design.

### Conclusion

To conclude, the use of telemedicine to facilitate and augment paediatric healthcare consultations is feasible and, in many cases, can work well with appropriate planning and quality facilities in place. HCPs and families who have experienced tele-consultations generally report high satisfaction and usability for such services. However, telemedicine is not practical for every clinical situation (such as cases where complex physical examinations or specific physical therapies are needed or a parent cannot articulate a child's condition), and its implementation can create an array of obstacles for healthcare workers in providing care to their full potential. Well-designed studies, undertaken throughout the implementation process are needed, in addition to a comprehensive systematic review of academic databases and grey literature, to establish the evidence base for user experiences of implementing paediatric telemedicine. Notwithstanding, our review will assist HCPs with the knowledge and information necessary to optimise clinical care safely through telemedicine *in situ*ations where normal clinical services are interrupted or reduced. Further reviews with more refined and focused research settings and exhaustive literature searches are warranted. A visual summary of our findings and conclusions is available in Supplementary Image 2.

## Data Availability Statement

The original contributions generated in the study are included in the article/Supplementary Material, further inquiries can be directed to the corresponding author.

## Author Contributions

GO'M, LT, and JS designed the study. LC, NA, and LT screened abstracts, titles, and full texts. LT extracted data and completed critical appraisal/risk of bias. LC and NA verified 20% of extracted data and completed critical appraisal for 20% of studies to ensure consistency. LT, GO'M, and JPM drafted and finalised the manuscript with critical feedback from JS, NA, and LC. All authors contributed to the article and approved the submitted version.

## Conflict of Interest

The authors declare that the research was conducted in the absence of any commercial or financial relationships that could be construed as a potential conflict of interest.

## Abbreviations

HCP: Healthcare Professional
ICT: Information and Communication Technology
MMAT: Mixed-Methods Appraisal Tool
CFIR: Consolidated Framework for Implementation Research.
