# Supplementary material for: Barriers and Facilitators for Implementing Paediatric Telemedicine: Rapid Review of User Perspectives
Source: Front Pediatr. 2021 Mar 17;9:630365. doi: 10.3389/fped.2021.630365 (PMC8010687; doi:10.3389/fped.2021.630365)
Supplement: Supplementary file 1 [file Table_1.DOCX]

| **Included studies must be:** | **Studies will be excluded if they are:** |
| --- | --- |
| 1. Qualitative, quantitative, mixed-methods studies examining use of telemedicine consultations for paediatric care | 1. Assessing the use of a technology with a primary purpose other than for facilitating communication between or with health professionals for paediatric care |
| 1. Assessments of telemedicine undertaken in a clinical setting with staff who are medical/allied health professionals | 1. Case studies (i.e. one patient) or documents such as reviews or commentaries, that do not report empirical findings |
| 1. Studies that report issues relating to implementation of telemedicine, including barriers/facilitators for introducing telemedicine, attitudes, experiences or satisfaction among users (staff or patients). | 1. Studies that do not report issues related to implementation (such as those reporting only clinical outcomes or those reporting economic evaluations). |

## Additional file 2: Inclusion & exclusion criteria

*Studies published in a language other than English or published prior to 2005 were excluded.*
