# Supplementary material for: Barriers and Facilitators for Implementing Paediatric Telemedicine: Rapid Review of User Perspectives
Source: Front Pediatr. 2021 Mar 17;9:630365. doi: 10.3389/fped.2021.630365 (PMC8010687; doi:10.3389/fped.2021.630365)
Supplement: Supplementary file 2 [file Table_2.DOCX]

| Additional file 3: Included studies & characteristics | | | | | | |
| --- | --- | --- | --- | --- | --- | --- |
| Author | **Year** | **Paediatric field** | **Telemedicine use** | **Participants** | **Design** | **Analysis** |
| Bator et al | 2015 | Surgical clinic | Anticipated | Patient/families (n=1032) | Quantitative descriptive | Descriptive statistics, chi squared test |
| Brova et al | 2018 | Emergency medicine | Experienced | Providers (n=107) | Quantitative descriptive | Descriptive statistics, chi-squared or Wilcoxon rank sum test |
| Bullock et al | 2017 | Rheumatology | Anticipated | Patient/family (n=159)  Child age (years): <6 (9%), 6-12 (41%), ≥13 (48%) | Quantitative descriptive | Descriptive statistics, Kruskal-Wallis, and Fisher's exact test |
| Cady et al | 2015 | Multi-morbidities | Experienced | Patient/family (n=139)  Child age (years): 2-5 (42%), 6-12 (42%), 13-15 (15%) | Randomised controlled trial | ANOVA/ ANCOVA, Fisher's exact, McNemar's; Open-ended feedback analysis not described |
| Coker et al | 2019 | Psychology | Experienced | Patient/family (n=342)  Child age (years): mean 8.6 (SD ± 2.3 ) | Randomised controlled trial | Regression analyses, intention to treat |
| DeAntonio et al | 2019 | Surgical clinic | Experienced | Patient/family (n=24)  Child age (years): mean 7.6 (SD ± 6.0) | Quantitative descriptive | Descriptive statistics |
| Fang et al | 2018 | Neonatology | Experienced | Healthcare providers (physicians and nurses): Survey (n=111) & focus groups (n=49) | Mixed methods | Descriptive statistics, thematic analysis |
| Fefferman et al | 2016 | Radiology | Experienced | Providers (n=16) | Quantitative descriptive | Not described |
| Fieleke et al | 2008 | Dermatology | Experienced | Providers (n=54) | Quantitative descriptive | Descriptive statistics |
| Greenberg et al | 2006 | Psychiatry | Experienced | Providers (Five focus groups n=6-9) + Families (interviews n=12)  Child age (years): mean 9.3 (range 4-14) | Qualitative | Thematic analysis |
| Haimi et al | 2018 | General paediatrics | Experienced | Providers: Physicians (n=15) | Qualitative | Thematic analysis |
| Hopper et al | 2011 | Genetic services | Experienced | Patient/family (n=10)  Child age (years): 8-14 | Quantitative descriptive | Descriptive statistics |
| Karlsudd et al | 2008 | Multi-disciplinary care for functional disabilities | Experienced | Patient/family (n=30) and providers (n=17) | Mixed methods | Descriptive statistics, interview analysis not described |
| Kessler et al | 2016 | Rheumatology | Anticipated | Parents/guardians: n=256  *Child age not presented* | Quantitative descriptive | Descriptive statistics, chi-squared, McNemar’s tests and Wilcoxon rank sum test |
| Kruger et al | 2011 | General paediatrics | Experienced | Healthcare providers: (n=33 consultants) | Mixed methods | Not described |
| Lai et al | 2018 | Primary care/multiple paediatric specialties | Experienced | Providers (n=388) | Quantitative descriptive | Descriptive statistics |
| Marconi et al | 2014 | Emergency medicine | Experienced | Patient/family (n=100)  Child age (years): mean 5.99 (SD ± 4.3) | Quantitative non-randomised | Summary statistics |
| McConnochie et al | 2010 | Primary care | Experienced | Providers (n=47) | Quantitative descriptive | Descriptive statistics |
| McConnochie et al | 2005 | General paediatrics | Experienced | Patient/family (n=229)  Child age (years): mean 4.0 | Quantitative descriptive | Descriptive statistics |
| McCrossan et al | 2014 | Cardiology | Experienced | Providers (n=35) | Quantitative descriptive | Descriptive statistics and Spearman’s rank correlation coefficient |
| Qubty et al | 2018 | Neurology | Experienced | Patient/family (n=51)  Child age (years): mean 5.6 (SD ± 3.5) | Quantitative descriptive | Descriptive statistics |
| Ray et al | 2017 | General paediatrics | Anticipated | Patients (n=2) and carers (n=19)  Child age (years): 0-4 (29%), 5-12 (14%), 13-17 (38%), 18-21 (19%) | Qualitative | Thematic analysis |
| Russo et al | 2017 | General paediatrics | Anticipated | Patient/family (n=751)  Child age (years): 0-5 (47%), 6-10 (27%), 11-17 (22%), ≥18 (5%) | Quantitative descriptive | Descriptive statistics, univariate and multi-variable logistic regression |
| Seckeler et al | 2015 | Cardiology | Experienced | Providers (n=65) | Quantitative descriptive | Descriptive statistics |
| Smith et al | 2014 | Surgical clinic | Experienced | Healthcare providers: Telehealth coordinators (n=2) and surgeons (n=6) | Mixed methods | Not described |
| Uscher-Pines et al | 2014 | Emergency medicine | Both | Healthcare providers (n=25) | Mixed methods | Descriptive statistics, Thematic analysis |
| Zachariah et al | 2012 | General paediatrics | Experienced | Providers (n=7) | Quantitative descriptive | Descriptive, chi-squared test |
