# Supplementary material for: Barriers and Facilitators for Implementing Paediatric Telemedicine: Rapid Review of User Perspectives
Source: Front Pediatr. 2021 Mar 17;9:630365. doi: 10.3389/fped.2021.630365 (PMC8010687; doi:10.3389/fped.2021.630365)
Supplement: Supplementary file 3 [file Table_3.DOCX]

| Additional file 4: Critical Appraisal Results using MMAT | | | | | | | | |
| --- | --- | --- | --- | --- | --- | --- | --- | --- |
| **Qualitative studies** | | | | | | | | |
| **Author** | **S1** | **S2** | **1.1** | **1.2** | **1.3** | **1.4** | **1.5** |  |
| Greenberg | Yes | Yes | Yes | Yes | Yes | Yes | Yes |  |
| Haimi | Yes | Yes | Yes | Can't tell | Yes | Yes | Can't tell |  |
| Ray | Yes | Yes | Yes | Yes | Yes | Yes | Yes |  |
| **Randomised controlled trials** | | | | | | | | |
| **Author** | **S1** | **S2** | **2.1** | **2.2** | **2.3** | **2.4** | **2.5** |  |
| Coker | Yes | Yes | Yes | Yes | Can't tell | No | Yes |  |
| Cady | Yes | Yes | Yes | Yes | No | No | Yes |  |
| **Quantitative non-randomised studies** | | | | | | | | |
| **Author** | **S1** | **S2** | **3.1** | **3.2** | **3.3** | **3.4** | **3.5** |  |
| Marconi | Yes | Yes | Yes | Yes | Yes | Yes | Yes |  |
| **Quantitative descriptive studies** | | | | | | | | |
| **Author** | **S1** | **S2** | **4.1** | **4.2** | **4.3** | **4.4** | **4.5** |  |
| DeAntonio | Yes | Yes | Yes | No | Yes | Yes | No |  |
| Bator | Yes | Yes | Yes | Yes | Yes | No | Yes |  |
| Brova | Yes | Yes | Yes | No | Can't tell | Yes | Yes |  |
| Bullock | Yes | Yes | Yes | No | Can't tell | Yes | Yes |  |
| Fefferman | Yes | Yes | Yes | Can't tell | Can't tell | Can't tell | Yes |  |
| Fieleke | Yes | Yes | Yes | Can't tell | Can't tell | No | Yes |  |
| Hopper | Yes | Yes | Yes | No | Can't tell | No | Yes |  |
| Kessler | Yes | Yes | Yes | Can't tell | Can't tell | Can't tell | Yes |  |
| Lai | Yes | Yes | Yes | Yes | Can't tell | Yes | Yes |  |
| McConnochie (2010) | Yes | Yes | Yes | Can't tell | Can't tell | Can't tell | Yes |  |
| McCrossan | Yes | Yes | Yes | Yes | Can't tell | Yes | Yes |  |
| Qubty | Yes | Yes | Yes | Can't tell | Can't tell | No | Yes |  |
| Russo | Yes | Yes | Yes | Can't tell | Can't tell | No | Yes |  |
| Seckeler | Yes | Yes | Yes | Can't tell | Can't tell | No | Yes |  |
| Zachariah | Yes | Yes | Can't tell | Can't tell | Can't tell | No | Yes |  |
| McConnochie (2005) | Yes | Yes | Yes | Yes | Can't tell | Can't tell | Yes |  |
| **Mixed methods studies** | | | | | | | | |
| **Author** | **S1** | **S2** | **5.1** | **5.2** | **5.3** | **5.4** | **5.5** |  |
| Fang | Yes | Yes | Yes | Yes | Yes | Yes | No |  |
| Kruger | No | Can't tell | Yes | No | Can't tell | Can't tell | Can't tell |  |
| Smith | Yes | Yes | Yes | Yes | Can't tell | No | Can't tell |  |
| Uscher-Pines | Yes | Yes | No | Yes | Yes | Yes | No |  |
| Karlsudd | Yes | Yes | Yes | Can't tell | Can't tell | Can't tell | Can't tell |  |
