# Supplementary figures and images for: Barriers and Facilitators for Implementing Paediatric Telemedicine: Rapid Review of User Perspectives
Source: Front Pediatr. 2021 Mar 17;9:630365. doi: 10.3389/fped.2021.630365 (PMC8010687; doi:10.3389/fped.2021.630365)

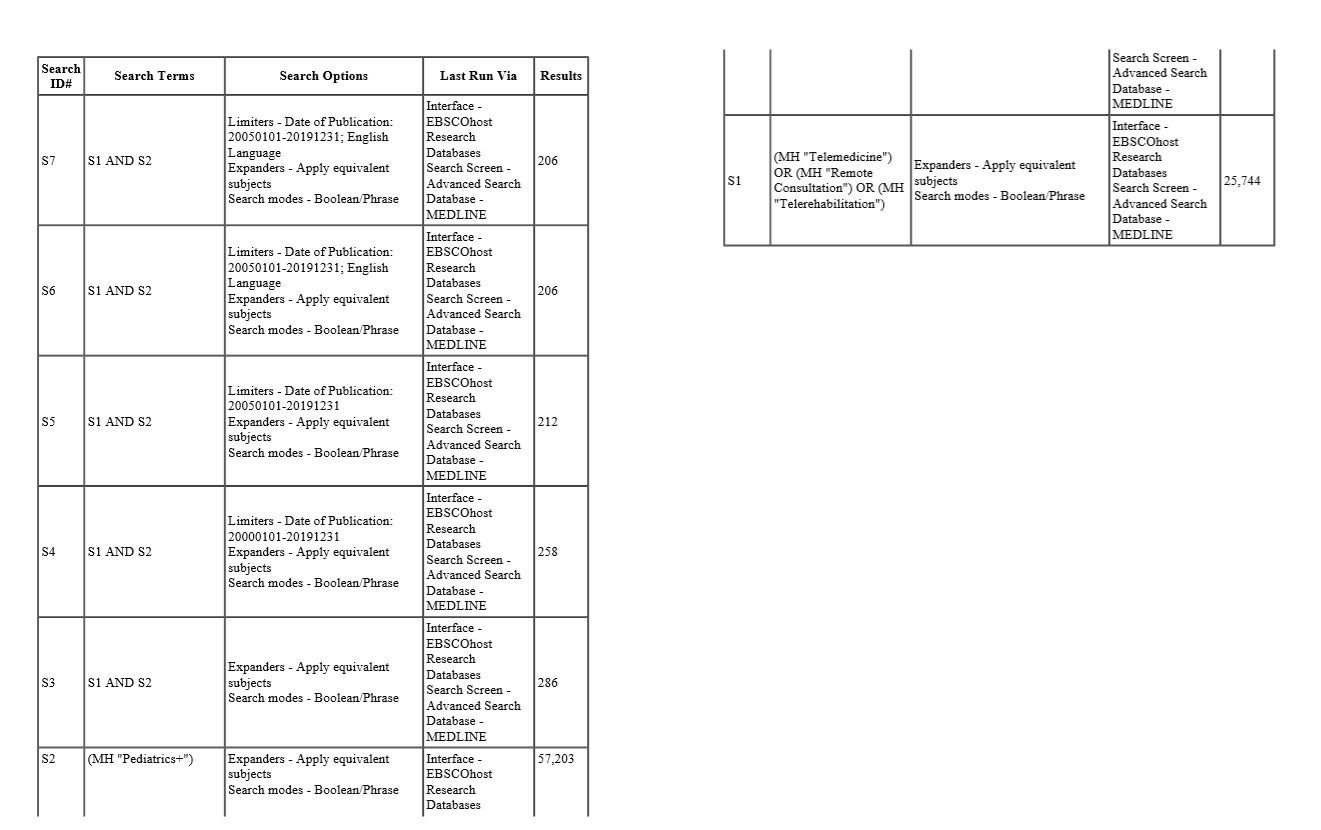

Supplement: Supplementary file 4 [file Image_1.PNG]
