# Supplementary material for: Barriers and Facilitators for Implementing Paediatric Telemedicine: Rapid Review of User Perspectives
Source: Front Pediatr. 2021 Mar 17;9:630365. doi: 10.3389/fped.2021.630365 (PMC8010687; doi:10.3389/fped.2021.630365)
Supplement: Supplementary file 5 [file Image_2.pdf]

Implementing paediatric telemedicine

Rapid evidence synthesis

COVID-19 has created a need for remote working and social distancing wherever possible. The use of video consultations (telemedicine) has become invaluable for healthcare practitioners in maintaining contact with patients and families. Implementation of telehealthcare is also a key action point of the Department of Health's eHealth Strategy for Ireland. We undertook a rapid review of the literature to summarise the evidenced barriers and facilitators for implementing paediatric telemedicine. The purpose was to inform and equip those tasked with implementing electronic health policies locally to predict and overcome issues.

Key findings & recommendations

There is **generally high acceptability** among healthcare practitioners (HCPs) and families who have experience using it. Families who live greater distances from clinics find it especially useful.

All end users should be involved in implementation (administrative staff, HCPs, families) to ensure the process is well planned. The goals of the service should be defined and disseminated clearly from the outset. Staff are more likely to buy in to the service if they are **clear about its purpose.**

Telemedicine will not lend itself well to every type of appointment or suit every family, and **cannot replace in-person care.** If a child is experiencing symptoms and cannot articulate these, it can create a major challenge in the absence of a physical examination.

Safety (risk assessment) and medico-legal concerns are primary sources of worry for HCPs with regards use of telemedicine, highlighting the need for robust protocols.

Many HCPs have low proficiency in technology and **worries about technical problems** are also common. Comprehensive training and continuous, accessible technical support is vital. A suitable designated area for tele-consultations is also very important.

There is **a need for well established workflow plans** for not only tele-consultations but the administrative work and personnel needed to facilitate these. This will avoid confusion and/or uneven workload.

How did we identify these?

Because the situation with COVID-19 was changing rapidly, and restrictions on movement were already in place, we wanted to use evidence to inform the implementation of telemedicine within Children's Health Ireland at Temple Street. However, as no evidence syntheses were available, there was a clear need for a review of this issue in the context of child health. Given the urgency of the situation, we conducted a rapid review, by focusing our search on one primary academic database to produce a timely review.

1. We undertook a mixed methods systematic review in May 2020, by first searching for studies in the MEDLINE academic database

2. We checked more than 200 articles for any information relevant to assisting implementation of paediatric telemedicine

3. We assessed the quality of 27 eligible studies, then summarised all relevant barriers and facilitators for incorporating telemedicine to paediatrics

Tully, L., Case, L., Arthurs, N., Sorensen, J., Marcin, JP., & O'Malley, G. 2021. Barriers and facilitators for implementing paediatric telemedicine: rapid review of user perspectives. Front. Pediatr. doi: 10.3389/fped.2021.630365

RCSI UNIVERSITY OF MEDICINE AND HEALTH SCIENCES

Children's Health Ireland at Temple Street

SPHeRE Structured Population and Health-services Research Education

HRB Health Research Board
